# Halogen Bonding in Sulphonamide Co-Crystals: X···π Preferred over X···O/N?

**DOI:** 10.3390/molecules28155910

**Published:** 2023-08-06

**Authors:** Tobias Heinen, Sarah Merzenich, Angelina Kwill, Vera Vasylyeva

**Affiliations:** Laboratory for Molecular Crystal Engineering, Department of Inorganic and Structural Chemistry, Heinrich-Heine University Duesseldorf, Universitaetstr. 1, 40225 Dusseldorf, Germany; t.heinen@hhu.de (T.H.);

**Keywords:** crystal engineering, pharmaceutical co-crystals, halogen bonding, halogen-π interactions, non-covalent interactions

## Abstract

Sulphonamides have been one of the major pharmaceutical compound classes since their introduction in the 1930s. Co-crystallisation of sulphonamides with halogen bonding (XB) might lead to a new class of pharmaceutical-relevant co-crystals. We present the synthesis and structural analysis of seven new co-crystals of simple sulphonamides N-methylbenzenesulphonamide (NMBSA), N-phenylmethanesulphonamide (NPMSA), and N-phenylbenzenesulphonamide (BSA), as well as of an anti-diabetic agent Chlorpropamide (CPA), with the model XB-donors 1,4-diiodotetrafluorobenzene (14DITFB), 1,4-dibromotetrafluorobenzene (14DBTFB), and 1,2-diiodotetrafluorobenzene (12DITFB). In the reported co-crystals, X···O/N bonds do not represent the most common intermolecular interaction. Against our rational design expectations and the results of our statistical CSD analysis, the normally less often present X···π interaction dominates the crystal packing. Furthermore, the general interaction pattern in model sulphonamides and the CPA multicomponent crystals differ, mainly due to strong hydrogen bonds blocking possible interaction sites.

## 1. Introduction

Sulphonamides are one of the essential pharmaceutical compound classes [1,2,3,4]. Over 5000 derivatives have been investigated for pharmaceutical applications, and 70 are used today. One example is sulfamidochrysoidine (trade name Prontosil), the first sulphonamide with pharmaceutical use and one of the first synthetic antibacterial drugs. It was discovered in 1932, reported in 1935, and honored with the Nobel Prize in medicine in 1939 [5,6]. Since then, sulphonamide drugs have been used in a wide range of applications, among others in antibacterial drugs [7,8,9,10], anti-diabetic agents [11,12,13,14,15], antiretroviral drugs [16,17,18,19], nonsteroidal anti-inflammatory drugs [20,21,22], and cardiac medications [23,24,25].

Like many other modern pharmaceuticals, some sulphonamides lack bioavailability [26,27]. One well-documented way to overcome this problem and simultaneously improve other macroscopic properties is co-crystallisation [28,29,30,31]. By profoundly investigating interaction patterns, general patterns of sulphonamides might be found. This is based on the well-known synthon theory, which despite its failures is still the starting point of rational design and could lead not only to one co-crystal but to a whole new class of pharmaceutical-relevant co-crystals.

Halogen bonds (XBs), an attractive interaction of a halogen moiety’s (-Cl, -Br, -I) partially positive charged σ-hole with a partially negative charged area, is along with hydrogen bonding one of the most important anisotropic intermolecular interactions [32]. There are numerous XBs; for example, strong XBs are formed with ions and oxygen and nitrogen moieties. XBs formed between two halogens are categorised into Type I and II based on the geometrical parameters [33]. In some cases, the so-called Quasi Type I/II can occur [34]. Some rare examples of XBs with aromatic π-systems have been reported [35,36,37,38]. These X···π interactions are weaker than the others mentioned because of the relatively widespread electron density along the π-system.

**Scheme 1:**
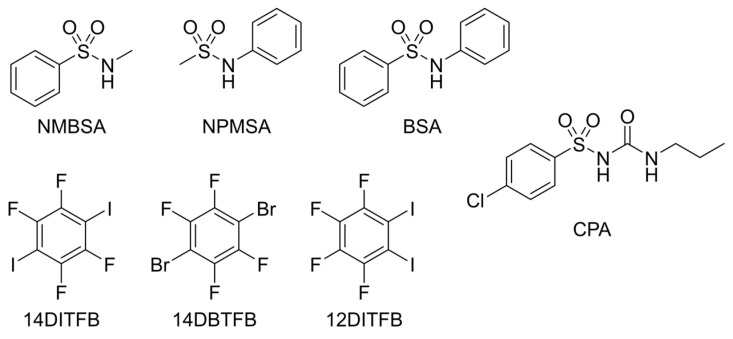
Molecular structures of used substances.

This work presents a series of rationally designed co-crystalline systems of selected sulphonamides with halogen-bonding donors (Scheme 1). Selected survey objects range from the simplest archetypal sulphonamides to their pharmaceutically active derivative CPA. The simplest sulphonamides are N-methylbenzenesulphonamide (NMBSA) [39], N-phenylmethanesulphonamide (NPMSA) [40,41], and N-phenylbenzenesulphonamide (BSA) [42]. They are derivates only substituted by methyl and phenyl moieties, which are nonpolar and sterically manageable. On the other hand, there is Chlorpropamide (CPA) [12], which has been used as an anti-diabetic agent. CPA is more complex and belongs to the sulphonamide subcategory sulphonylureas, which inhibit a urea-like moiety and are used to treat diabetes. It has six polymorphic modifications [43,44] and one known co-crystal with 4,4′-dipyridyl [45]. The coformers, 1,4-diiodotetrafluorobenzene (14DITFB), 1,4-dibromotetrafluorobenzene (14DBTFB), and 1,2-diiodotetrafluorobenzene (12DITFB), are model XB coformers used in various studies to obtain multicomponent systems. Based on the obtained crystal structures, a topological analysis of intermolecular interactions with a primary emphasis on different types of halogen bonding is carried out.

## 2. Results

### 2.1. Crystal Structure of NMBSA-14DITFB (1:1), ***1***

**Figure 1:**
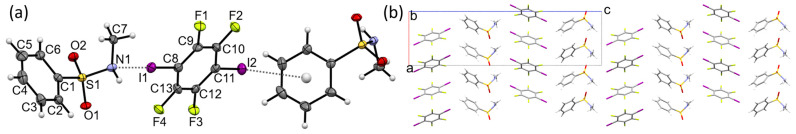
(**a**) ORTEP plot of 1 with occurring halogen bonds I1···N1 (3.057(6) Å) and I2···π_cg_ (4.045 Å), where labelled atoms are part of the asymmetric unit; (**b**) structure along the *b*-axis highlighting the alternating layers of NMBSA and 14DITFB.

The NMBSA-14DITFB (1:1) co-crystal, **1**, crystallises in the orthorhombic space group *Pna*2_1_ with NMBSA and 14DITFB in a 1:1 ratio (Figure 1a). These form alternating layers along the *ab*-plane in a simple ABAB motif (Figure 1b). The sulphonamide layer is interconnected via strong and weak hydrogen bonds. Predominantly, a chain motif is established between the amine and the sulphone group (d(O2···H1) = 2.1(1) Å, d(O2···N1) = 3.009(7) Å). The 14DITFB layers are only loosely connected with weak F···π interactions in a herringbone pattern. The layers interact via two independent halogen bonds. On one side, there is an XB (C8-I1···N1) with the free nitrogen electron pair. It is short (d = 3.057(6) Å) and straight (∢ = 179.8(2)°), both of which are signs of strong interactions. On the other side is a halogen bond between iodine and the π-system. It is relatively weak with a distance of d(I2···cg) = 4.045 Å and an angle of ∢(C11-I2···cg) = 166.0°.

### 2.2. Crystal Structure of NPMSA-14DITFB (1:1), ***2***

**Figure 2:**
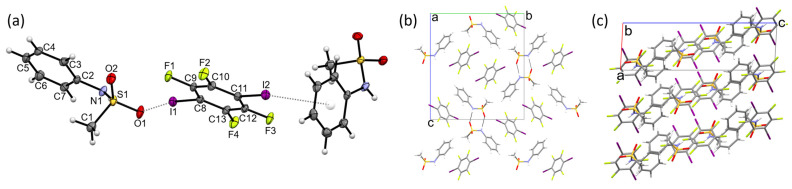
(**a**) ORTEP plot of **2** with occurring halogen bonds I1···O1 (2.994(2) Å) and I2···π_cg_ (3.535 Å), where labelled atoms are part of the asymmetric unit; (**b**) crystal packing along the *a*-axis highlighting the NPMSA hydrogen-bonded dimers; (**c**) layers interconnected via halogen bonding.

The NPMSA-14DITFB (1:1) co-crystal, **2**, crystallises in a monoclinic space group *P*2_1_*/n* with NPMSA and 14DITFB in a 1:1 ratio (Figure 2a). The sulphonamide forms a dimer with its inverted counterpart, utilising strong HB between the sulphone and amine group (d(O1···H1) = 2.15(4) Å, d(O1···N1) = 2.933(3) Å) with the inversion centre in the middle. Four dimers are aligned in channels along the *a*-axis, including two 14DITFB molecules (Figure 2b). The 14DITFB interacts on one site via a strong XB with the sulphone group (d(I1···O1) = 2.994(2) Å; d(I1···O2) = 3.496(2) Å) and on the other one with the π-system of the phenyl ring (d(I2···cg) = 3.536 Å, ∢(C11-I2···cg) = 154.1°), similarly to structure **1**. The XBs connect the individual layers of which this structure is constructed. The layers are oriented along the (101) plane (Figure 2c).

### 2.3. Crystal Structure of NPMSA-14DITFB (2:1), ***3***

**Figure 3:**
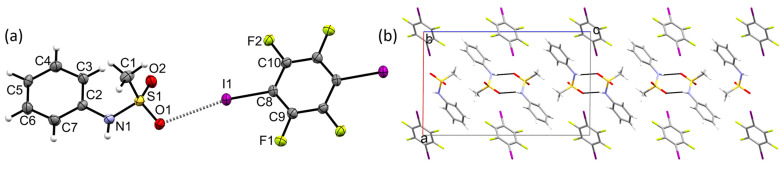
(**a**) ORTEP plot of **3** with occurring halogen bonds I1···O1 (3.089(3) Å), where labelled atoms are part of the asymmetric unit; (**b**) structure along the *a*-axis highlighting the NPMSA hydrogen-bonded dimers.

The NPMSA-14DITFB (2:1) co-crystal, **3**, crystallises in a monoclinic space group *P*2_1_*/c* with NPMSA and 14DITFB in a 2:1 ratio (Figure 3a). The structure is not only related to **2** by its components but also by important structural elements. It forms almost identical sulphonamide HB dimers (d(O1···H1) = 2.19(5) Å, d(O1···N1) = 2.982(4) Å) and I···O_2_S- XBs (d(I1···O1) = 3.089(3) Å; d(I1···O2) = 3.751(2) Å). However, due to the different stoichiometry, and an additional inversion centre present in 14DITFB, it forms only one distinctive XB. This is also reflected in both smaller asymmetric unit and unit cell. Nevertheless, system **3** could not be reproduced in subsequent crystallisation attempts after the initial production.

### 2.4. Crystal Structure of BSA-14DITFB (2:1), ***4***

**Figure 4:**
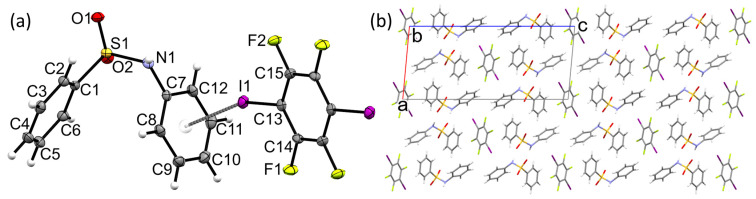
(**a**) ORTEP plot of **4** with occurring halogen bonds I1···cg (3.461 Å), where labelled atoms are part of the asymmetric unit; (**b**) crystal packing along the *b*-axis highlighting the interaction network.

The BSA-14DITFB (2:1) co-crystal, **4**, crystallises in a monoclinic space group *P*2_1_/*c* with BSA and 14DITFB in a 2:1 ratio (Figure 4a). The BSA builds a 2D net pattern in the *ab*-plane using strong and weak HBs. Chains are based on strong O···H-N HB (d(O1···H1) = 2.08(3) Å; d(O1···N1) = 2.864(2) Å). These chains are interacting via weak O···H-C HB (d(O2···H5) = 2.62(2) Å; d(O2···C5) = 3.409(2) Å). The 14DITFB interacts with the π-systems linking the nets via XB. This XB is the shortest X···cg interaction presented in this publication, with a distance of 3.461 Å. The angle towards the centre of the aromatic system is ∢(C13-I1···cg) = 162.1°.

### 2.5. Crystal Structure of CPA-14DITFB (2:1), ***5***

**Figure 5:**
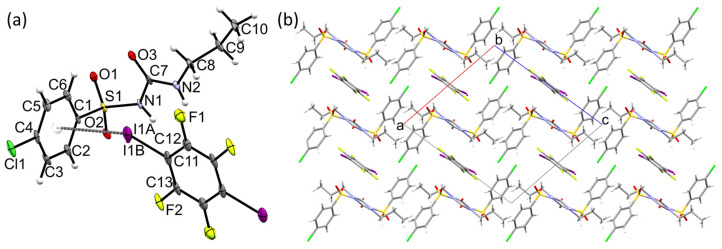
(**a**) ORTEP plot of **5** with occurring halogen bonds I1···cg (3.626 Å), where labelled atoms are part of the asymmetric unit; (**b**) crystal packing along the *b*-axis highlighting the CPA layers interconnected by 14DITFB.

The CPA-1,4DITFB (2:1) co-crystal, **5**, crystallises in a monoclinic space group, *P*2_1_*/n*, with CPA and 14DITFB in a 2:1 ratio (Figure 5a). The strongest intermolecular interaction within this structure is the HB chain pattern of the CPA molecules. The molecules’ alignment regarding each other is defined by the 2_1_-screw axis going through the urea moiety of the oxygen double bond. The hydrogen atoms of the urea moiety interact with the oxygen atom of the next urea moiety, (d(H1···O3) = 1.91(4) Å, d(H2A···O3) = 2.21(4) Å), and one of the oxygen atoms of the sulphone group (d(H2A···O1) = 2.36(4) Å). These chains are interconnected via π-stacking and form zig-zag-planes. The planes are connected via 14DITFB. In the middle of 14DITFB is an inversion centre; therefore, only half of the molecule is part of the asymmetric unit. The XB distance is relatively long (d(I1A···cg) = 3.626 Å, ∢(C12-I1A···cg) = 151.6°).

The structure was solved with a disordered iodine atom (87:13). The disorder is based on different possible interactions with the π-system. Only the iodine atom was refined separately since the whole molecule would have required a lot of restrains.

### 2.6. Crystal Structure of CPA-14DBTFB (2:1), ***6***

**Figure 6:**
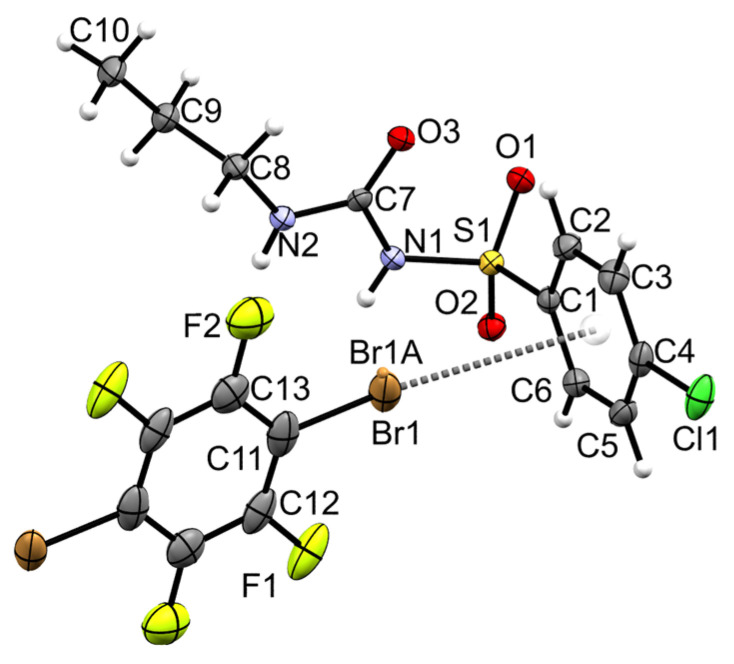
ORTEP plot of **6** with occurring halogen bonds I1···cg (3.639 Å), where labelled atoms are part of the asymmetric unit.

The crystal structures of CPA-14DBTFB (2:1), **6** (Figure 6), and **5** are isostructural, which is a common phenomenon for 14DITFB and 14DBTFB co-crystals [26]. Unsurprisingly, the distances hardly change. For the halogen-π XB (d(Br1··· cg) = 3.639 Å, ∢(C13-Br1···cg) = 173.0°), caused by the smaller bromine, this results in an even weaker intermolecular interaction.

The structure was solved with a disordered bromine atom (53:47). The disorder is based on different possible interactions with the π-system, and the higher disorder here compared to **5** is a direct consequence of the weaker XBs. Only the bromine atom was refined separately since a refinement of the whole molecule requires a lot of restrains, although in this case, the ellipsoids of F1 and F2 also indicate disorder.

### 2.7. Crystal Structure of CPA-12DITFB (2:1), ***7***

**Figure 7:**
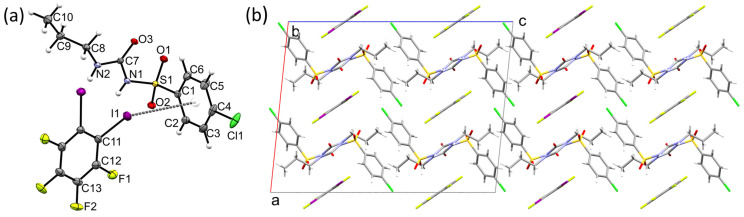
(**a**) ORTEP plot of **7** with occurring halogen bonds I1···cg (4.168 Å), where labelled atoms are part of the asymmetric unit; (**b**) crystal packing along the *b*-axis highlighting the CPA layers interconnected by 12DITFB.

The CPA-12DITFB (2:1) co-crystal, **7**, crystallises in a monoclinic space group *C*2*/c* with CPA and 12DITFB in a 2:1 ratio (Figure 7). Strong and weak HBs hold the CPA zig-zag-plane structure together, and π-stacking is the same as in the crystal structures **5** and **6**. In contrast, here the space between the layers is filled with 12DITFB, which interacts via weak XBs with the π-system of CPA (d(I1···cg) = 4.168 Å, ∢(C11-I1···cg) = 155.4°). The 12DITFB is locked in the centre in place by the rotational axis and cannot move in any way without losing symmetry. Therefore, it cannot move closer towards the π-systems of two CPA molecules of the lattice structure. Again, the long XBs result from symmetry-related needs and the slight geometrical mismatch.

## 3. Discussion

**Figure 8:**
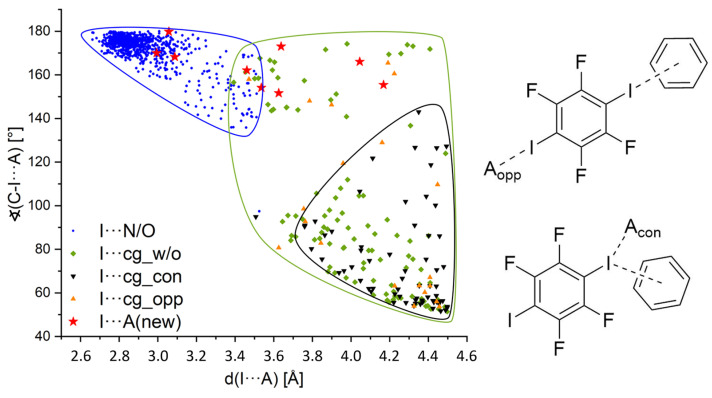
Scatterplot for different types of halogen bonds of 12DITFB, 13DITFB, and 14DITFB with halogen bond acceptors (A) plotted over distance (d) in Angström [Å] and angle (∢) in degree [°]. A total of 1010 interactions are plotted: 807 with strong acceptors in blue points (I···O 224, I···N 583), 101 in green routes without any interaction with N/O (I···cg_w/o), 70 in black triangles pointing down for concurrent interactions of N/O and cg (I···cg_con), 23 orange triangles pointing up for opposing interactions of N/O and cg (I···cg_opp), and 9 red stars for the new interactions presented in this study (I···A).

To give a broader context for the described XB patterns, in-depth research on intermolecular XB interactions of the three diiodotetrafluorobenzenes 12DITFB, 13DITFB, and 14DITFB in the Cambridge Structural Database (CSD) [46] has been performed. Therefore, we analysed 553 structures and categorised the halogen bonds into four major groups, which are discussed in the following section. The results are presented in Figure 8; all search parameters are listed in the Supplementary Materials.

In the blue area, a total of 807 halogen bonds are depicted (583 I···N, 224 I···O). This group is the by far most populated one, which is hardly discernible since most of the points are overlapping. These interactions are the classic strong XBs, which are well-known and obviously often described [32]. Therefore, these interactions are the ones we expected for our systems.

The area of XB interactions with aromatic systems is more extensive and diverse. Therefore, to increase the comparability, only C6 aromatic systems were considered. The distance (d), as well as the angle (∢), is measured relative to the centre of gravity (cg) for the same reason. The broadest category includes solely the I···cg interactions, without any XBs with a nitrogen or an oxygen (I···cg_w/o) atom being involved. The 101 I···cg interactions reach from 3.4 Å to 4.5 Å and from 50° to 180°. A cut-off was made at 4.5 Å since interactions at this point are almost impossible. The subgroup I···cg_opp within this category contains 23 XB interactions of DITFBs, which on the opposing iodine are interacting with nitrogen or oxygen. We had the hypothesis that they might behave with less direction and be more focused on the I···O/N site; hence, the stronger interaction with the electron-rich moieties should dominate the interaction pattern. But, interestingly, based on the scatterplot, no difference is noticeable compared to the I···cg_w/o, and the interactions are distributed in the whole green area. In contrast to that, I···cg_con indicates interactions where the same iodine entity shares both I···O/N and I···cg. From the 70 XB interactions found in the database, 62 are mostly caused by symmetry between “con” and “opp”. However, this subgroup, as indicated by the black line, is almost solely found in the region with longer distances and a lower angle. This is understandable considering the competing strong XB acceptor. Overall, the ratio between I···O/N and I···cg given in the literature is 4:1 (807:194). On the other hand, a more rigorous view on I···cg would move the ratio even further in favour of I···O/N.

The nine XBs in the presented structures **1**–**7** are plotted as red stars in the scatterplot above and summarised in Table 1 together with the van der Waals radii (vdW) [47] for respective interactions. Three of these XBs are I···O/N and fit very well into analogue literature interactions (blue area). The remaining six interactions are I···cg_w/o or I···cg_opp interactions (green area). They are within the expected area, but all of them have a relatively high angle, and the XBs from **2**–**6** also have a relatively short interaction distance.

Within this study, the co-crystallisation experiments with the basic sulphonamides were performed first, resulting in structures **1**–**4**. These structures share I···O/N and I···cg equally, slightly overrepresenting the latter compared to the literature. Co-crystallisation experiments on a real-world example, namely CPA, followed to elucidate if the same result will occur for a more complex sulphonamide system. Interestingly, a strong I···O/N interaction was not formed in either of the resulting structures, **5**–**7**. At this point, we began an in-depth analysis of the structures to clarify why the lessons learned from the small-model molecules are not transferable to the larger one and if there is a reason why I···cg might be stronger than I···O/N.

Newly synthesised compounds **2** and **3** both consist of the same entities of NPMSA and 14DITFB in different ratios, a phenomenon, which does not occur often for co-crystals. In some sense, it can be seen as a polymorphic behaviour. Similar to known examples of disappearing polymorphs [48,49], it was impossible to reproduce **3** in any possible way, which shows that it is disfavoured. Both structures have an intermolecular interaction with the sulphone oxygen moiety, but while in **3** both iodines of the 14DITFB are symmetry-equivalent, in **2** the second iodine interacts with a π-system. This comes from the significantly stronger I···O. It is likely that in the process of lattice formation, after forming this bond, it is sterically hindered since it needs to be elongated and rotated relative to the sulphone moiety. Therefore, the next-best option is forming an XB with the π-system favoured, leading to structure **2**.

In contrast, co-crystal structures of CPA (**5**–**7**) show only XBs of the I···cg; CPA forming zig-zag-planes which are intercalated with the halogen bond donors naturally occurs for these structures. The backbone of these planes are strong hydrogen bond chains of the urea moiety, which are well known in the literature [50,51,52]. However, the intermediate spaces seem to be somewhat larger than ideal for the halo-benzenes. For structures **5** and **6** it results in a longer I···cg bond than necessary, since the 14DITFB and 14DBTFB are secured by symmetry. For **7** the situation is slightly different. The iodines are not in a para but in an ortho position, and the aromatic centres are in a far-from-ideal position, resulting in the longest XB (d(I1···cg) = 4.168 Å).

Let us go back to the initial thought that XBs in sulphonamide systems might prefer π-systems over O/N-moieties as acceptors. More realistically, the HBs are stronger and, therefore, the predominant building blocks are either catameric structures (**1**, **4**–**7**) or dimeric structures (**2**, **3**). The halogens take what was left, following Ostwald’s rule of stages [53]. So XBs with π-systems have become a common interaction within the investigated structures against our expectations based on statistical knowledge and model sulphonamide co-crystal structures.

## 4. Materials and Methods

### 4.1. Synthesis of NMBSA-14DITFB (1:1), ***1***

Single crystals of **1** suitable for SCXRD were synthesised by dissolving NMBSA (10 mg, 58 µmol) and 14DITFB (12 mg, 30 µmol) in 1 mL of chloroform. The solution evaporated slowly at room temperature to give clear, colourless, plate-shaped crystals. The pure phase was obtained by neat-grinding NMBSA (45 mg, 261 µmol) and 1,4DITFB (105 mg, 263 µmol) in an MM 400 ball mill from Retsch with 20 Hz for 20 min.

### 4.2. Synthesis of NPMSA-14DITFB (1:1), ***2***

Single crystals of **2** suitable for SCXRD were obtained by dissolving NPMSA (10 mg, 58 µmol) and 14DITFB (12 mg, 30 µmol) in 1 mL of acetonitrile. The solution rapidly evaporated at room temperature to give clear, colourless, plate-shaped crystals. The pure phase was then obtained by neat-grinding NMBSA (40 mg, 234 µmol) and 14DITFB (94 mg, 234 µmol) in an MM 400 ball mill from Retsch with 20 Hz for 30 min.

### 4.3. Synthesis of NPMSA-14DITFB (2:1), ***3***

Pure-phase and single crystals of **3** suitable for SCXRD were synthesised by dissolving NPMSA (10 mg, 58 µmol) and 14DITFB (12 mg, 30 µmol) in 1 mL of acetonitrile. Clear, colourless block-shaped crystals were obtained after several days via slow evaporation at room temperature. Phase **3** could not be reproduced under the same or several different conditions. All attempts led to either **2** or a mixture of the base components.

### 4.4. Synthesis of BSA-14DITFB (2:1), ***4***

Single crystals of **4** suitable for SCXRD were synthesised by dissolving BSA (10 mg, 42 µmol) and 14DITFB (9 mg, 22 µmol) in 1 mL of ethanol. The solution evaporated slowly at room temperature for several days to form clear, colourless block-shaped crystals. The pure phase was additionally obtained by neat-grinding BSA (80 mg, 343 µmol) and 14DITFB (69 mg, 172 µmol) in an MM 400 ball mill from Retsch with 20 Hz for 20 min.

### 4.5. Synthesis of CPA-14DITFB (2:1), ***5***

Clear, colourless block-shaped single crystals of **5** suitable for SCXRD were synthesised by dissolving CPA (45 mg, 163 µmol) and 14DITFB (65 mg, 163 µmol) in 1 mL of methanol. The solution rapidly evaporated at room temperature. The pure phase was obtained via liquid-assisted grinding CPA (270 mg, 976 µmol) with 14DITFB (196 mg, 488 µmol) and 20 µL of methanol in an MM 400 ball mill from Retsch with 20 Hz for 20 min.

### 4.6. Synthesis of CPA-14DBTFB (2:1), ***6***

Single crystals of **6** suitable for SCXRD were synthesised by dissolving CPA (39 mg, 1000 µmol) and 14DBTFB (70 mg, 1672 µmol) in 1 mL of methanol, followed by rapid evaporation at room temperature. Clear, colourless block-shaped crystals were formed overnight. The pure phase was obtained via liquid-assisted grinding CPA (260 mg, 940 µmol), 14DBTFB (145 mg, 470 µmol), and 20 µL methanol in an MM 400 ball mill from Retsch with 20 Hz for 20 min.

### 4.7. Synthesis of CPA-12DITFB (2:1), ***7***

Single crystals of **7** suitable for SCXRD were synthesised by fast solution evaporation of CPA (45 mg, 163 µmol) and 12DITFB (65 mg, 163 µmol) dissolved in 1 mL of methanol at room temperature. Clear, colourless block-shaped crystals were formed overnight. The pure phase was obtained by liquid-assisted grinding CPA (500 mg, 1806 µmol), 1,2DITFB (363 mg, 1806 µmol), and 20 µL methanol in an MM 400 ball mill from Retsch with 20 Hz for 20 min. 

### 4.8. SCXRD

Single Crystal X-ray Diffraction of **1**–**7**: Suitable single crystals were selected and mounted with silicon oil on a cryo-loop. Diffraction data were recorded with a Rigaku XtaLAB Synergy S diffractometer with a Hybrid Pixel Array Detector. Diffraction data were recorded with ω-scans using a micro-focus sealed X-ray tube PhotonJet X-ray Source (Cu (λ= 1.54184 Å) or Mo (λ = 0.71073 Å)), mirror monochromator. Cell refinement, data reduction, and absorption correction were executed with CrysAlisPro [54]. OLEX2 [55] was used to solve the crystal structures with SHELXS and refine it with SHELXL [56]. All non-hydrogen positions were refined with anisotropic displacement parameters. Hydrogens were positioned geometrically with U_iso_(H_CH(aliph.)_) = 1.5 U_eq_ and U_iso_(H_CH(arom.)_) = 1.2 U_eq_, except for amide hydrogens, which were positioned and refined freely. The crystallographic data for structures **1**–**7** have been deposited at the Cambridge Crystallographic Data Centre (CCDC 2258821-2258827). Important crystallographic data and refinement parameters for systems **1**–**7** are listed in Appendix A, Table A1, Table A2, Table A3, Table A4, Table A5, Table A6 and Table A7. Figures were prepared with Mercury software (2022.3.0) [57].

### 4.9. PXRD

Powder X-ray Diffraction measurements were performed on a Rigaku Miniflex diffractometer in θ/2θ geometry from 5° to 50° at ambient temperature using Cu Kα radiation (λ = 1.54182 Å) and a rotating sample holder. Simulations were carried out with Mercury software [57]. All recorded PXRDs, including their comparison with the simulated ones, are available in the Supplementary Materials.

## 5. Conclusions

This study has presented four new co-crystal structures of archetypal sulphonamides (**1**–**4**) and three co-crystal structures of the pharmaceutically used CPA (**5**–**7**). All presented structures exhibit halogen bonds with para- or ortho-substituted halogen benzene derivatives, but against prior expectations based on statistical analysis and model sulphonamides, X···O/N was not formed in CPA multicomponent systems, but X···π XBs were. This is not a sign of XBs favouring aromatic systems over these strong Lewis bases but rather a consequence of competing hydrogen bonds (HBs). The sulphonamides formed strong HB dimers and catamers with O/N, interacting with multiple sites. Therefore, XBs fall behind and interact with the aromatic π-systems instead. In addition, some X···π XBs are unusually long, which is caused by symmetry and rigid sulphonamide grids.

## Figures and Tables

**Table 1:** Comparison of structural elements within structures **1**–**7** as well as van der Waals (vdW) radii. Distances d in [Å], angles ∢ in [°]; measurements involving the centre of a phenyl group are labelled with cg.

	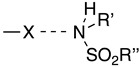	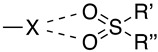	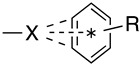
**1**	d = 3.057(6), ∢ = 179.8(2)	-	d_cg_ = 4.045, ∢_cg_ = 166.0
**2**	-	d = 2.994(2), ∢ = 170.01(8)	d_cg_ = 3.536, ∢_cg_ = 154.1
**3**	-	d = 3.088(3), ∢ = 168.2(1)	-
**4**	-	-	d_cg_ = 3.461, ∢_cg_ = 162.1
**5**	-	-	d_cg_ = 3.626, ∢_cg_ = 151.6
**6**	-	-	d_cg_ = 3.638, ∢_cg_ = 173.0
**7**	-	-	d_cg_ = 4.168, ∢_cg_ = 155.4
vdW radii [47]	d(I···N) = 3.53	d(I···O) = 3.50	d(I···C) = 3.68, d(Br···C) = 3.53

## Data Availability

Not applicable.

## Supplementary Materials

The following supporting information can be downloaded at: https://www.mdpi.com/article/10.3390/molecules28155910/s1. Table S1: hydrogen-bond geometry for NMBSA-14DITFB (1:1), **1**.; Table S2: halogen-bond geometry for NMBSA-14DITFB (1:1), **1**; Table S3: hydrogen-bond geometry for NPMSA-14DITFB (1:1), **2**; Table S4: halogen-bond geometry for NPMSA-14DITFB (1:1), **2**; Table S5: hydrogen-bond geometry for NPMSA-14DITFB (2:1), **3**; Table S6: halogen-bond geometry for NPMSA-14DITFB (2:1), **3**; Table S7: hydrogen-bond geometry for BSA-14DITFB (2:1), **4**; Table S8: halogen-bond geometry for BSA-14DITFB (2:1), **4**; Table S9: hydrogen-bond geometry for CPA-14DITFB (2:1), **5**; Table S10: halogen-bond geometry for CPA-14DITFB (2:1), **5**; Table S11: hydrogen-bond geometry for CPA-14DBTFB (2:1), **6**; Table S12: halogen-bond geometry for CPA-14DBTFB (2:1), **6**; Table S13: hydrogen-bond geometry for CPA-12DITFB (2:1), **7**; Table S14: halogen-bond geometry for CPA-12DITFB (2:1), **7**; Figure S1: PXRDs of **1** as-synthesised (a.s.) and simulated (sim.) based on the single-crystal structure; Figure S2: PXRDs of **2** as-synthesised (a.s.) and simulated (sim.) based on the single-crystal structure; Figure S3: PXRDs of **3** as-synthesised (a.s.) and simulated (sim.) based on the single-crystal structure; Figure S4: PXRDs of **4** as-synthesised (a.s.) and simulated (sim.) based on the single-crystal structure; Figure S5: PXRDs of **5** as-synthesised (a.s.) and simulated (sim.) based on the single-crystal structure; Figure S6: PXRDs of **6** as-synthesised (a.s.) and simulated (sim.) based on the single-crystal structure; Figure S7: PXRDs of **7** as-synthesised (a.s.) and simulated (sim.) based on the single crystal structure; List of CSD Search Parameters.

## Appendix A

**Table A1:** Crystallographic data and refinement details for structure **1**.

Structure	1
Empirical formula	C_13_H_9_F_4_I_2_NO_2_S
Moiety formula	SO_2_NC_7_H_9_, C_6_F_4_I_2_
Formula weight [g/mol]	573.07
Temperature [K]	100(1)
Crystal system	orthorhombic
Space group	*Pna*2_1_
a, b, c [Å]	8.96170(10), 5.82430(10), 32.1685(5)
α, β, γ [°]	90, 90, 90
Volume [Å^3^]	1679.06(4)
Z	4
ρ_calc_ [g/cm^3^]	2.267
μ [mm^−1^]	31.045
F(000)	1072.0
Crystal size [mm]	0.14 × 0.07 × 0.03
Radiation	CuK*α* (λ = 1.54184 Å)
2Θ range for data collection [°]	5.494 to 157.628
Index ranges	−11 ≤ h ≤ 10, −7 ≤ k ≤ 6, −40 ≤ l ≤ 40
Reflections collected	17831
Independent reflections	3313 (R_int_ = 0.0479, R_sigma_ = 0.0356)
Data/restraints/parameters	3313/2/213
Goodness of fit on F^2^	1.085
Final R indexes (I ≥ 2σ (I))	R_1_ = 0.0328, wR_2_ = 0.0871
Final R indexes (all data)	R_1_ = 0.0335, wR_2_ = 0.0876
Largest diff. peak/hole [e Å^−3^]	1.27/−1.02
Flack parameter	−0.021(8)

**Table A2:** Crystallographic data and refinement details for structure **2**.

Structure	2
Empirical formula	C_13_H_9_F_4_I_2_NO_2_S
Moiety formula	SO_2_NC_7_H_9_, C_6_F_4_I_2_
Formula weight [g/mol]	573.07
Temperature [K]	100(1)
Crystal system	monoclinic
Space group	P2_1_/n
a, b, c [Å]	5.6059(2), 16.1462(4), 18.2847(4)
α, β, γ [°]	90, 93.083(2), 90
Volume [Å^3^]	1652.63(8)
Z	4
ρ_calc_ [g/cm^3^]	2.303
μ [mm^−1^]	3.979
F(000)	1072.0
Crystal size [mm]	0.15 × 0.08 × 0.04
Radiation	MoK*α* (λ = 0.71073 Å)
2Θ range for data collection [°]	4.462 to 59.944
Index ranges	−7 ≤ h ≤ 7, −19 ≤ k ≤ 20, −25 ≤ l ≤ 25
Reflections collected	15,672
Independent reflections	3945 (R_int_ = 0.0314, R_sigma_ = 0.0259)
Data/restraints/parameters	3945/0/213
Goodness of fit on F^2^	1.038
Final R indexes (I ≥ 2σ (I))	R_1_ = 0.0227, wR_2_ = 0.0527
Final R indexes (all data)	R_1_ = 0.0275, wR_2_ = 0.0549
Largest diff. peak/hole [e Å^−3^]	0.72/−0.60

**Table A3:** Crystallographic data and refinement details for structure **3**.

Structure	3
Empirical formula	C_10_H_9_F_2_INO_2_S
Moiety formula	SO_2_NC_7_H_9_, 0.5(C_6_F_4_I_2_)
Formula weight [g/mol]	372.14
Temperature [K]	100(1)
Crystal system	monoclinic
Space group	P2_1_/c
a, b, c [Å]	11.4061(3), 5.74650(10), 18.4124(4)
α, β, γ [°]	90, 90.479(2), 90
Volume [Å^3^]	1206.80(5)
Z	4
ρ_calc_ [g/cm^3^]	2.048
μ [mm^−1^]	22.656
F(000)	716.0
Crystal size [mm]	0.12 × 0.07 × 0.05
Radiation	CuK*α* (λ = 1.54184 Å)
2Θ range for data collection [°]	7.752 to 153.698
Index ranges	−13 ≤ h ≤ 14, −4 ≤ k ≤ 6, −22 ≤ l ≤ 22
Reflections collected	8322
Independent reflections	2285 (R_int_ = 0.0386, R_sigma_ = 0.0328)
Data/restraints/parameters	2285/0/159
Goodness of fit on F^2^	1.072
Final R indexes (I ≥ 2σ (I))	R_1_ = 0.0272, wR_2_ = 0.0737
Final R indexes (all data)	R_1_ = 0.0293, wR_2_ = 0.0752
Largest diff. peak/hole [e Å^−3^]	0.64/−0.72

**Table A4:** Crystallographic data and refinement details for structure **4**.

Structure	4
Empirical formula	C_15_H_11_NO_2_F_2_SI
Moiety formula	SO_2_NC_12_H_11_, 0.5(C_6_F_4_I_2_)
Formula weight [g/mol]	434.21
Temperature [K]	100(1)
Crystal system	monoclinic
Space group	P2_1_/n
a, b, c [Å]	10.6190(2), 6.03240(10), 23.9571(6)
α, β, γ [°]	90, 95.244(2), 90
Volume [Å^3^]	1528.22(5)
Z	4
ρ_calc_ [g/cm^3^]	1.887
μ [mm^−1^]	2.259
F(000)	844.0
Crystal size [mm]	0.258 × 0.212 × 0.097
Radiation	MoK*α* (λ = 0.71073 Å)
2Θ range for data collection [°]	4.068 to 60.228
Index ranges	−14 ≤ h ≤ 14, −6 ≤ k ≤ 8, −32 ≤ l ≤ 28
Reflections collected	11,111
Independent reflections	3659 (R_int_ = 0.0273, R_sigma_ = 0.0269)
Data/restraints/parameters	3659/0/202
Goodness of fit on F^2^	1.057
Final R indexes (I ≥ 2σ (I))	R_1_ = 0.0203, wR_2_ = 0.0453
Final R indexes (all data)	R_1_ = 0.0222, wR_2_ = 0.0461
Largest diff. peak/hole [e Å^−3^]	0.43/−0.34

**Table A5:** Crystallographic data and refinement details for structure **5**.

Structure	5
Empirical formula	C_13_H_13_ClF_2_IN_2_O_3_S
Moiety formula	SO_3_N_2_C_10_ H_13_Cl, 0.5(C_6_F_4_I_2_)
Formula weight [g/mol]	477.66
Temperature [K]	100(1)
Crystal system	monoclinic
Space group	P2_1_/n
a, b, c [Å]	13.14950(10), 8.96850(10), 14.61480(10)
α, β, γ [°]	90, 102.8420(10), 90
Volume [Å^3^]	1680.43(3)
Z	4
ρ_calc_ [g/cm^3^]	1.888
μ [mm^−1^]	17.934
F(000)	932.0
Crystal size [mm]	0.51 × 0.18 × 0.18
Radiation	CuK*α* (λ = 1.54184 Å)
2Θ range for data collection [°]	10.256 to 158.07
Index ranges	−15 ≤ h ≤ 15, −10 ≤ k ≤ 10, −17 ≤ l ≤ 17
Reflections collected	24,561
Independent reflections	3342 (R_int_ = 0.0630, R_sigma_ = 0.0275)
Data/restraints/parameters	3342/24/227
Goodness of fit on F^2^	1.063
Final R indexes (I ≥ 2σ (I))	R_1_ = 0.0380, wR_2_ = 0.0998
Final R indexes (all data)	R_1_ = 0.0384, wR_2_ = 0.1002
Largest diff. peak/hole [e Å^−3^]	1.03/−2.26

**Table A6:** Crystallographic data and refinement details for structure **6**.

Structure	6
Empirical formula	C_13_H_13_BrClF_2_N_2_O_3_S
Moiety formula	SO_3_N_2_C_10_H_13_Cl, 0.5(C_6_Br_2_F_4_)
Formula weight [g/mol]	430.67
Temperature [K]	100(1)
Crystal system	monoclinic
Space group	P2_1_/n
a, b, c [Å]	13.1627(2), 8.95812(18), 14.5445(3)
α, β, γ [°]	90, 105.584(2), 90
Volume [Å^3^]	1651.94(6)
Z	4
ρ_calc_ [g/cm^3^]	1.732
μ [mm^−1^]	2.811
F(000)	860.0
Crystal size [mm]	0.4 × 0.1 × 0.1
Radiation	MoK*α* (λ = 0.71073 Å)
2Θ range for data collection [°]	4.878 to 52.742
Index ranges	−16 ≤ h ≤ 16, −11 ≤ k ≤ 11, −18 ≤ l ≤ 18
Reflections collected	44,260
Independent reflections	3378 (R_int_ = 0.0345, R_sigma_ = 0.0128)
Data/restraints/parameters	3378/33/227
Goodness of fit on F^2^	1.168
Final R indexes (I ≥ 2σ (I))	R_1_ = 0.0342, wR_2_ = 0.0712
Final R indexes (all data)	R_1_ = 0.0365, wR_2_ = 0.0722
Largest diff. peak/hole [e Å^−3^]	0.45/−0.37

**Table A7:** Crystallographic data and refinement details for structure **7**.

Structure	7
Empirical formula	C_13_H_13_ClF_2_IN_2_O_3_S
Moiety formula	SO_3_N_2_C_10_H_13_Cl, 0.5(C_6_F_4_I_2_)
Formula weight [g/mol]	477.66
Temperature [K]	100(1)
Crystal system	monoclinic
Space group	C2/c
a, b, c [Å]	16.9967(2), 8.90250(10), 22.2511(3)
α, β, γ [°]	90, 95.9650(10), 90
Volume [Å^3^]	3348.65(7)
Z	8
ρ_calc_ [g/cm^3^]	1.895
μ [mm^−1^]	2.231
F(000)	1864.0
Crystal size [mm]	0.51 × 0.326 × 0.206
Radiation	MoK*α* (λ = 0.71073 Å)
2Θ range for data collection [°]	4.82 to 60.264
Index ranges	−23 ≤ h ≤ 22, −12 ≤ k ≤ 12, −30 ≤ l ≤ 31
Reflections collected	41,319
Independent reflections	4452 (R_int_ = 0.0259, R_sigma_ = 0.0112)
Data/restraints/parameters	4452/0/217
Goodness of fit on F^2^	1.068
Final R indexes (I ≥ 2σ (I))	R_1_ = 0.0178, wR_2_ = 0.0440
Final R indexes (all data)	R_1_ = 0.0187, wR_2_ = 0.0444
Largest diff. peak/hole [e Å^−3^]	0.47/−0.57
