# Supplementary material for: Halogen Bonding in Sulphonamide Co-Crystals: X···π Preferred over X···O/N?
Source: Molecules. 2023 Aug 6;28(15):5910. doi: 10.3390/molecules28155910 (PMC10420850; doi:10.3390/molecules28155910)
Supplement: Supplementary file 1 [file molecules-28-05910-s001.zip › SI_molecules-2520932.pdf]

Table of Contents

Lists of Hydrogen bonds and halogen bonds in structures 1-7 2

Lists of PXRDs for Structures 1-7 5

List of CSD Search Parameters 9

Lists of Hydrogen bonds and halogen bonds in structures 1-7

NMBSA-14DITFB (1:1), 1

*Table S1: Hydrogen-bond geometry for NMBSA-14DITFB (1:1), **1**.*

D—H···A	D—H [Å]	d(H···A) [Å]	D(D···A) [Å]	∢(D—H···A) [°]
C6—H6···O1^{i}	0.95	2.59	3.284(9)	130
C7—H7B···F4^{ii}	0.98	2.58	3.474(9)	153
C7—H7C···O2^{iii}	0.98	2.60	3.287(9)	127
N1—H1···S1^{ii}	0.89(3)	2.91(7)	3.726(6)	153(12)
N1—H1···O2^{ii}	0.89(3)	2.14(5)	3.009(7)	164(14)

Symmetry codes: (i) x, y+1, z; (ii) x-1/2, -y+3/2, z; (iii) x-1/2, -y+5/2, z

*Table S2: Halogen-bond geometry for NMBSA-14DITFB (1:1), **1**.*

C—X···A	d(X···A) [Å]	∢(C—X···A) [°]
C8—I1···N1	3.057(6)	179.8(2)
C11—I2···C3^{i}	3.515(9)	173.6(2)
C11—I2···C4^{i}	3.476(9)	161.6(2)

Symmetry code: (i) 1-x,1-y,1/2+z

NPMSA-14DITFB (1:1), 2

*Table S3: Hydrogen-bond geometry for NPMSA-14DITFB (1:1), **2**.*

D—H···A	D—H [Å]	d(H···A) [Å]	D(D···A) [Å]	∢(D—H···A) [°]
C3—H3···O2	0.95	2.40	3.052(3)	126
C1—H1A···O2^{i}	0.98	2.44	3.373(4)	159
N1—H1···O1^{ii}	0.79(4)	2.15(4)	2.933(3)	169(4)

Symmetry codes: (i) x-1, y, z; (ii) -x, -y+1, -z+2

*Table S4: Halogen-bond geometry for NPMSA-14DITFB (1:1), **2**.*

C—X···A	d(X···A) [Å]	∢(C—X···A) [°]
C8—I1···O1	2.994(2)	170.02(8)
C8—I1···O2	3.496(2)	146.26(8)
C8—I1···S1	3.6892(7)	167.43(7)
C11—I2···C4^{i}	3.440(3)	172.08(9)
C11—I2···C5^{i}	3.557(3)	162.06(9)
C11—I2···cg^{i}	3.535	154.05
C11—I2···F2^{ii}	3.442(2)	114.87(8)

Symmetry code: (i) 1.5+x,1.5-y,1/2+z; (ii) 3-x,2-y,2-z

NPMSA-14DITFB (2:1), 3

*Table S5: Hydrogen-bond geometry for NPMSA-14DITFB (2:1), **3**.*

D—H···A	D—H [Å]	d(H···A) [Å]	D(D···A) [Å]	∢(D—H···A) [°]
C3—H3···O2	0.95	2.39	3.058(4)	127
N1—H1···O1^{i}	0.82 (5)	2.19(5)	2.982(4)	163(4)

Symmetry codes: (i) -x+1, -y+2, -z+1

*Table S6: Halogen-bond geometry for NPMSA-14DITFB (2:1), **3**.*

C—X···A	d(X···A) [Å]	∢(C—X···A) [°]
C8—I1···O1	3.089(3)	168.2(1)
C8—I1···O2*	3.751(2)	150.7(1)
C8—I1···S1*	3.8204(9)	170.5(1)

*Distances above sum of vdW radii but listed for comparison

BSA-14DITFB (2:1), 4

*Table S7: Hydrogen-bond geometry for BSA-14DITFB (2:1), **4**.*

D—H···A	D—H [Å]	d(H···A) [Å]	D(D···A) [Å]	∢(D—H···A) [°]
N1—H1···O1^{i}	0.79(3)	2.08(3)	2.864(2)	173(3)
C8—H8···O2^{ii}	0.95	2.43	3.293(2)	151

Symmetry codes: (i) -x+3/2, y+1/2, -z+3/2; (ii) x, y+1, z

*Table S8: Halogen-bond geometry for BSA-14DITFB (2:1), **4**.*

C—X···A	d(X···A) [Å]	∢(C—X···A) [°]
C13—I1···cg	3.461	162.09
C13—I1···C7	3.482(2)	169.15(6)
C13—I1···C8	3.546(2)	151.53(6)
C13—I1···C12	3.673(2)	168.23(6)

CPA-14DITFB (2:1), 5

*Table S9: Hydrogen-bond geometry for CPA-14DITFB (2:1), **5**.*

D—H···A	D—H [Å]	d(H···A) [Å]	D(D···A) [Å]	∢(D—H···A) [°]
N1—H1···O3^{i}	0.85(5)	1.90(4)	2.725(4)	162(5)
N2—H2A···O3^{i}	0.82(5)	2.20(4)	2.935(4)	151(5)
N2—H2A···O1^{i}	0.82(5)	2.35(4)	2.947(4)	131(5)

Symmetry codes: (i) -x+1/2, y+1/2, -z+3/2

*Table S10: Halogen-bond geometry for CPA-14DITFB (2:1), **5**.*

C—X···A	d(X···A) [Å]	∢(C—X···A) [°]
C11—I1A···cg*	3.625	151.55
C11—I1A···C4*	3.947(4)	148.4(1)
C11—I1A···C5*	3.764(4)	169.0(1)
C11—I1A···C6*	3.697(4)	162.5(1)

*Distances above sum of vdW radii but listed for comparison

CPA-14DBTFB (2:1), 6

*Table S11: Hydrogen-bond geometry for CPA-14DBTFB (2:1), **6**.*

D—H···A	D—H [Å]	d(H···A) [Å]	D(D···A) [Å]	∢(D—H···A) [°]
N1—H1···O3^{i}	0.79(4)	1.98(3)	2.733(2)	159(3)
N2—H2A···O3^{i}	0.80(3)	2.27(3)	2.981(2)	148(3)
N2—H2A···O1^{i}	0.80(3)	2.33(3)	2.948(2)	134(2)

Symmetry codes: (i) -x+1/2, y+1/2, -z+3/2

*Table S12: Halogen-bond geometry for CPA-14DBTFB (2:1), **6**.*

**C—X···A**	**d(X···A) [Å]**	**∢(C—X···A) [°]**
C11—Br1···cg*	3.639	173.03
C11—I1A···C1*	3.643(5)	164.1(2)
C11—I1A···C2*	3.852(5)	164.2(2)
C11—I1A···C6*	3.701(6)	157.2(2)

*Distances above sum of vdW radii but listed for comparison

CPA-12DITFB (2:1), 7

*Table S13: Hydrogen-bond geometry for CPA-12DITFB (2:1), **7**.*

**D—H···A**	**D—H [Å]**	**d(H···A) [Å]**	**D(D···A) [Å]**	**∢(D—H···A) [°]**
N1—H1···O3^{i}	0.79(2)	1.99(3)	2.743(1)	159(2)
N2—H2A···O3^{i}	0.81(2)	2.24(2)	2.962(1)	149(2)
N2—H2A···O1^{i}	0.81(2)	2.35(2)	2.963(2)	133(2)

Symmetry codes: (i) -x+1/2, y+1/2, -z+3/2

*Table S14: Halogen-bond geometry for CPA-12DITFB (2:1), **7**.*

**C—X···A**	**d(X···A) [Å]**	**∢(C—X···A) [°]**
C11—I1···cg	4.168	155.36
C11—I1···C1	4.221(1)	153.63(4)
C11—I1···C5	4.472(2)	162.82(4)
C11—I1···C6	4.311(2)	172.23(5)

*Distances above sum of vdW radii but listed for comparison

Lists of PXRDs for structures 1-7

NMBSA-14DITFB (1:1), 1

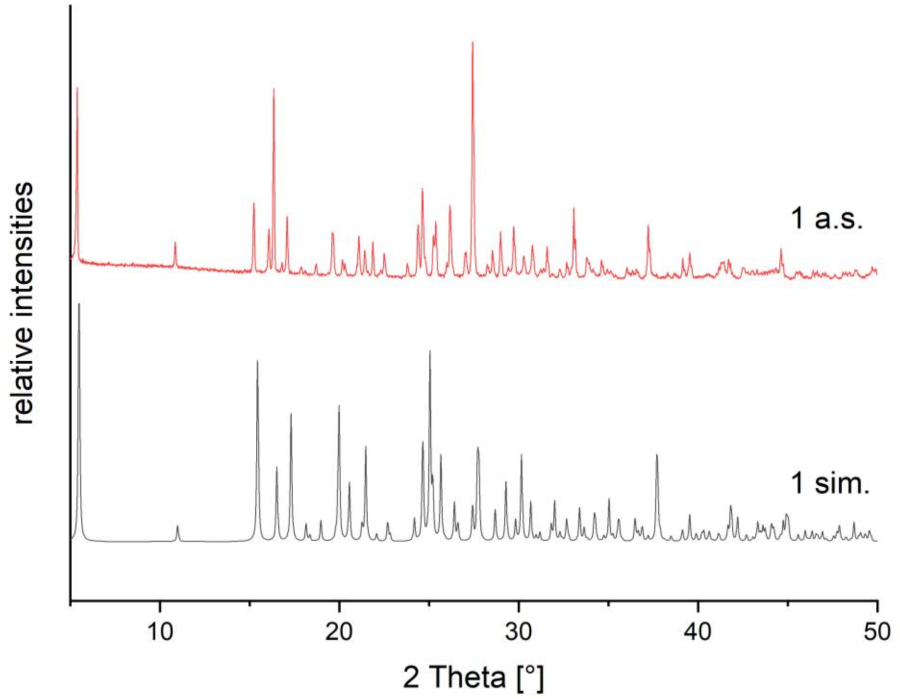

*Figure S1: PXRDs of **1** as synthesized (a.s.) and simulated (sim.) based on the single crystal structure.*

NPMSA-14DITFB (1:1), 2

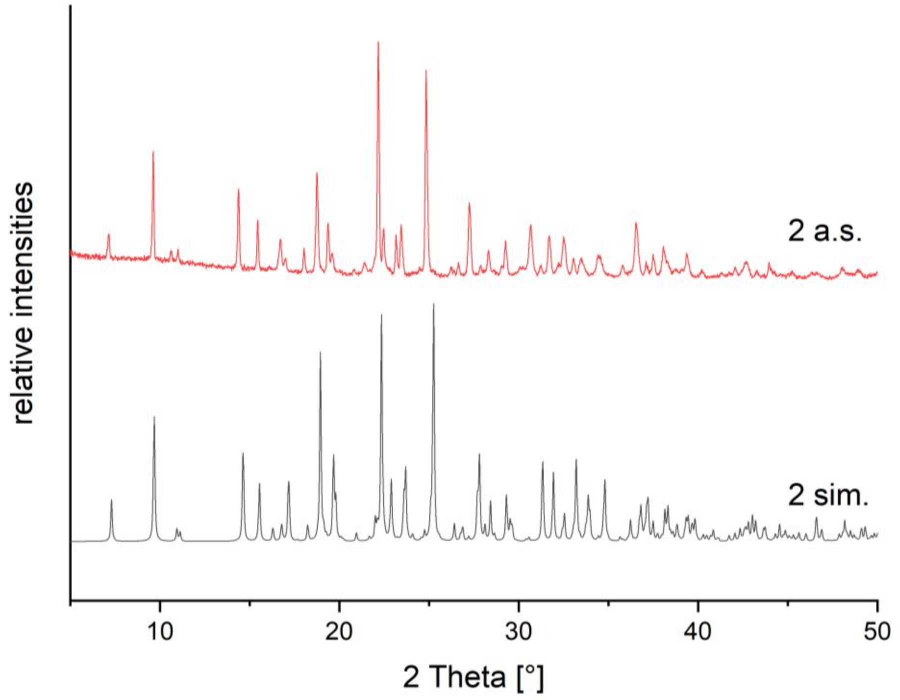

*Figure S2: PXRDs of **2** as synthesized (a.s.) and simulated (sim.) based on the single crystal structure.*

NPMSA-14DITFB (2:1), 3

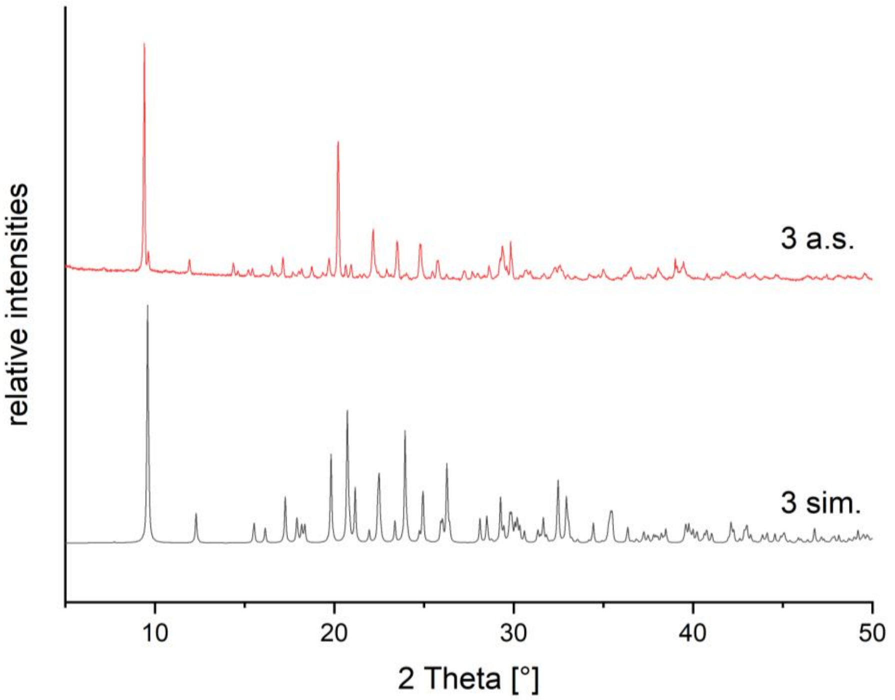

*Figure S3: PXRDs of **3** as synthesized (a.s.) and simulated (sim.) based on the single crystal structure.*

BSA-14DITFB (2:1), 4

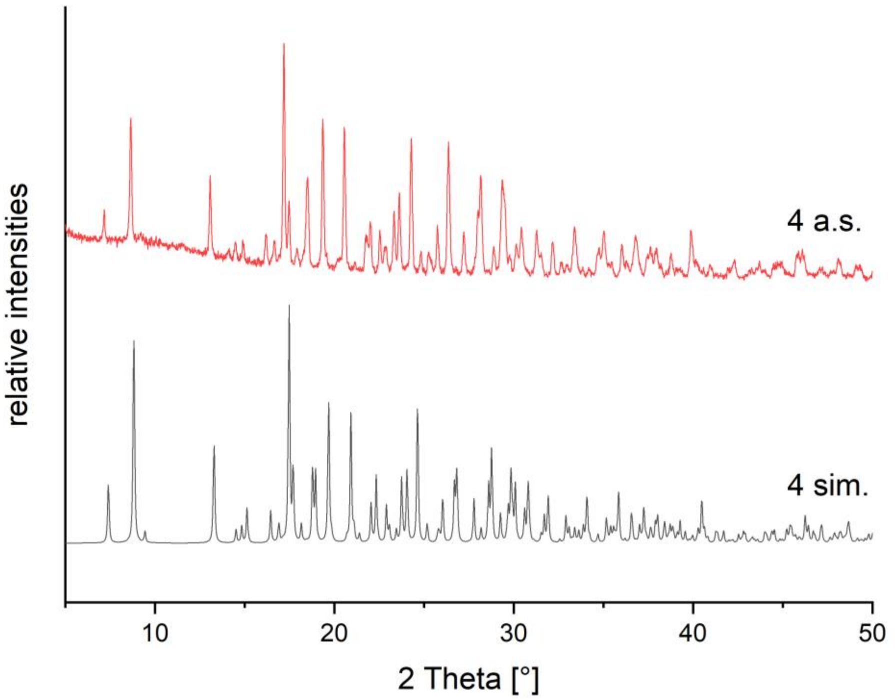

*Figure S4: PXRDs of **4** as synthesized (a.s.) and simulated (sim.) based on the single crystal structure.*

CPA-14DITFB (2:1), 5

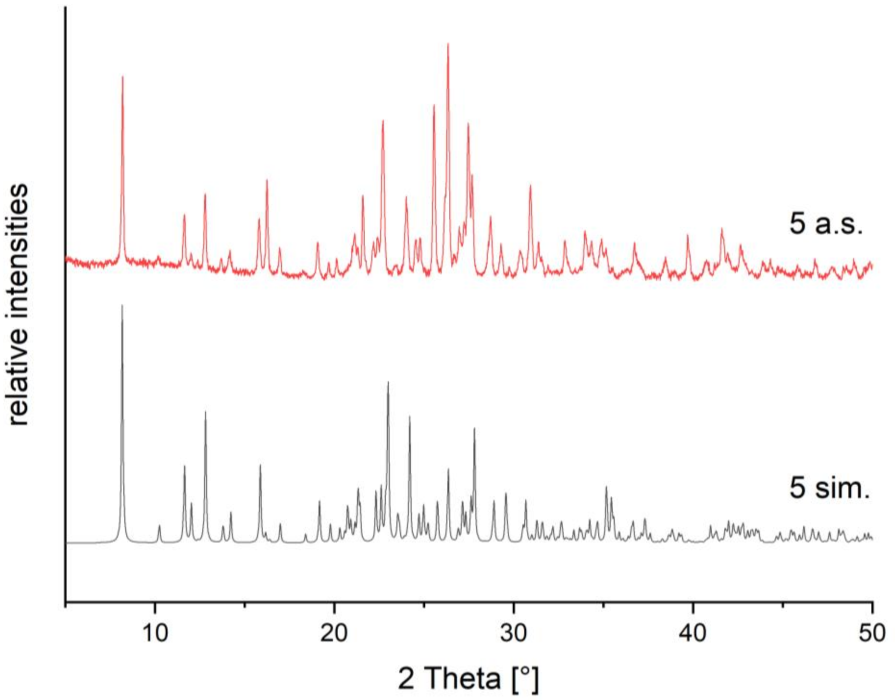

*Figure S5: PXRDs of **5** as synthesized (a.s.) and simulated (sim.) based on the single crystal structure.*

CPA-14DBTFB (2:1), 6

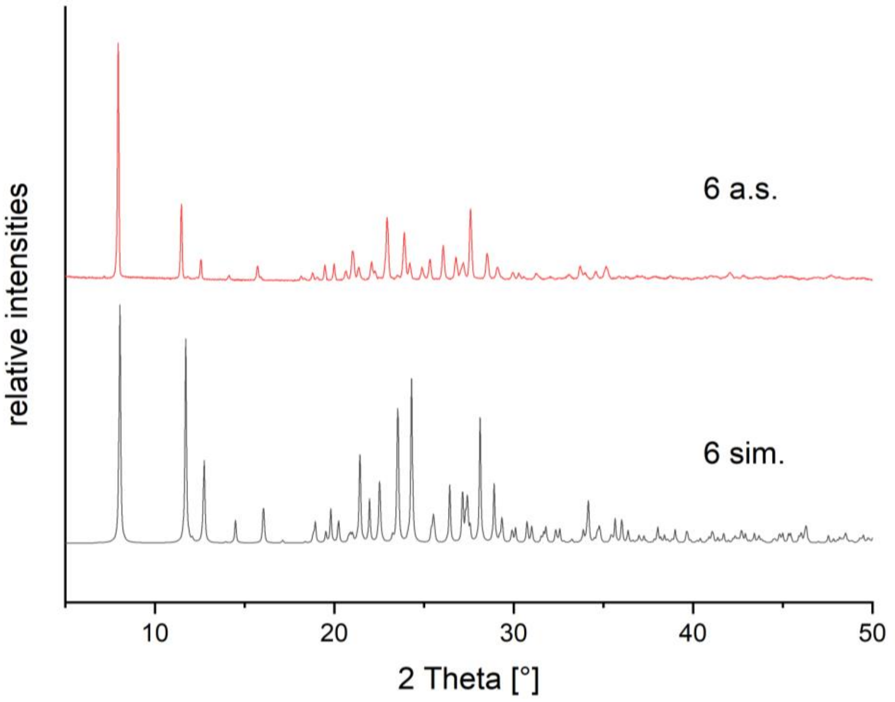

*Figure S6: PXRDs of **6** as synthesized (a.s.) and simulated (sim.) based on the single crystal structure.*

CPA-12DITFB (2:1), 7

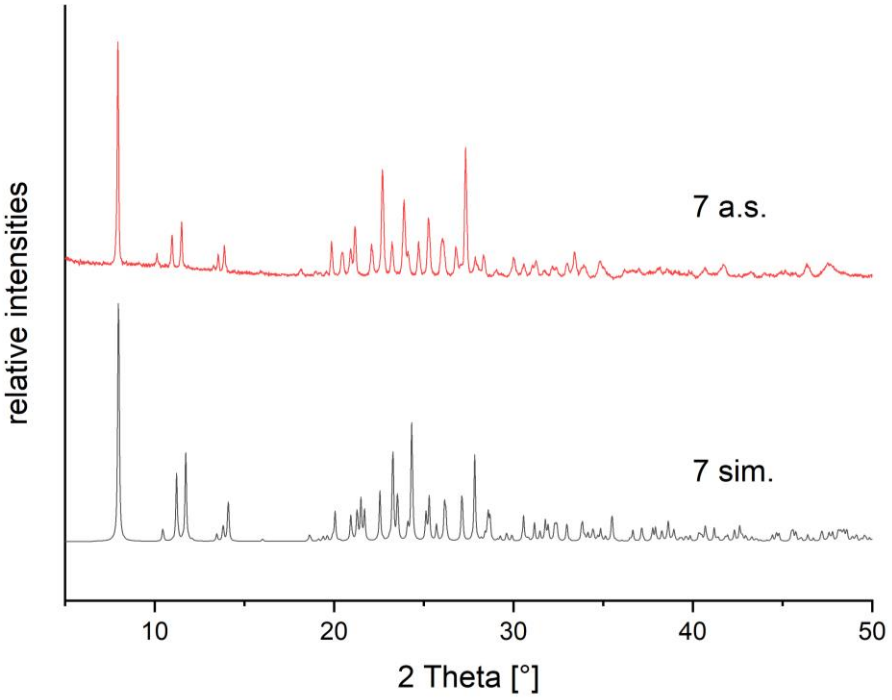

*Figure S7: PXRDs of **7** as synthesized (a.s.) and simulated (sim.) based on the single crystal structure.*

List of CSD Search Parameters

General Search Parameters

The search was performed in the CSD database (Update 11/12) with the *ConQuest* software. Following filters were used for every search request:

- 3D coordinates determined
- Only Non-disordered
- Only Single crystal structures
- R factor <= 0.1
- No errors
- No ions
- Only Organics

Subgroup DITFB_all

The subgroup of the diiodotetrafluorobenzenes (DITFB) – 1,4-diiodotetrafluorobenzene (14DITFB), 1,3-diiodotetrafluorobenzene (13DITFB), 1,2-diiodotetrafluorobenzene (12DITFB) – was defined by combining the following three queries with "OR" operator:

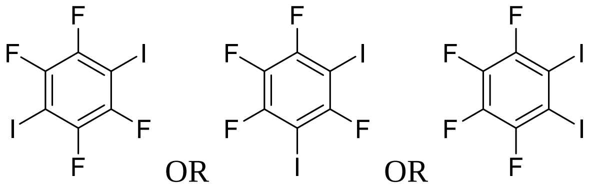

Figure S8: Searches for subgroup DITFB_all.

There are 553 structures which match this request and respectively in the according subgroup.

Search parameters for DITFB I···O interactions

Within the subgroup DITFB_all I···O interactions were searched with the following search request:

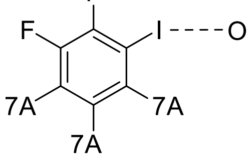

Figure S9: Search for I···O interactions. 7A stands for "Any Halogen".

The I···O contact is defined as "inter-molecular" and "distance within sum of VdW + 0.0 Å" (3.50 Å). A total of 224 interactions of this nature have been identified.

Search parameters for DITFB I···N interactions

Within the subgroup DITFB_all I···N interactions were searched with the following search request:

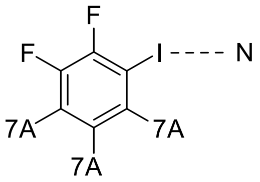

Figure S10: Search for I···N interactions. 7A stands for "Any Halogen".

The I···N contact is defined as "inter-molecular" and "distance within sum of VdW + 0.0 Å" (3.53 Å). A total of 583 interactions of this nature have been identified.

Search parameters for DITFB I···cg_w/o interactions

Within the subgroup DITFB_all I···cg interactions, which are not disturbed by a strong XB acceptor O or N by searching for the respective interaction and excluding all structures with I···O or I···N interactions:

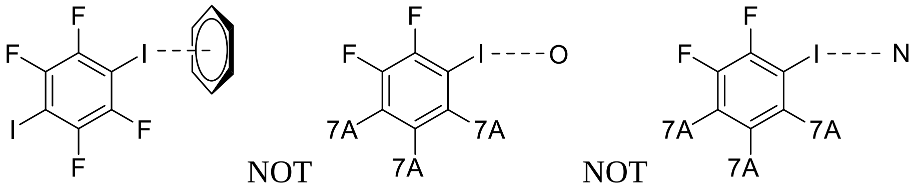

Figure S11: Search for I···cg_w/o interactions. 7A stands for "Any Halogen".

The I···cg contact is defined as "inter-molecular" and "distance range: 2.5 to 4.5 Å" A total of 101 interactions of this nature have been identified.

Search parameters for DITFB I···cg_con interactions

Within the subgroup DITFB_all I···cg interactions, which are concurrently occurring to an I···N or I···O interaction with the same iodine:

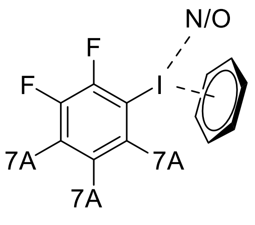

Figure S12: Search for I···cg_con interactions. 7A stands for "Any Halogen".

The I···cg contact is defined as "inter-molecular" and "distance range: 2.5 to 4.5 Å" A total of 70 interactions of this nature have been identified.

Search parameters for DITFB I···cg_opp interactions

Within the subgroup DITFB_all I···cg interactions, which are oppositely occurring to an I···N or I···O interaction with another iodine but not concurrently:

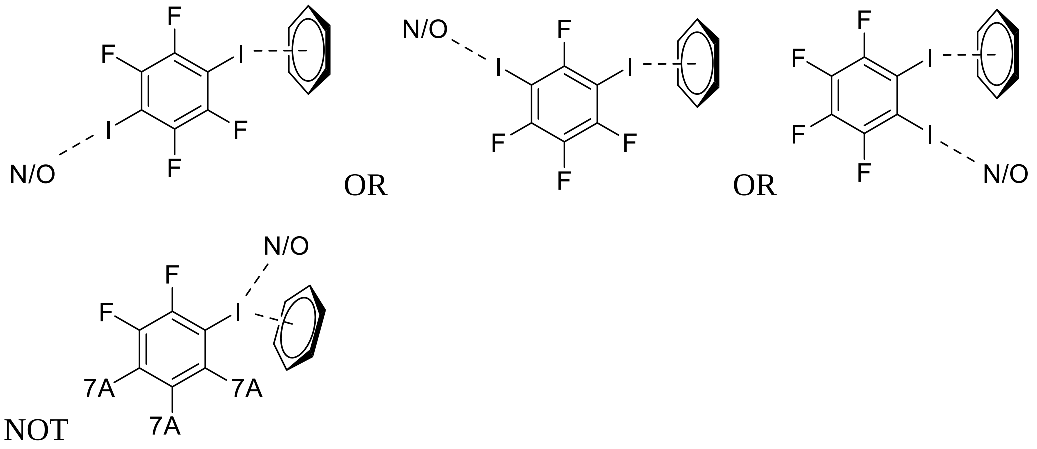

Figure S13: Search for I···cg_opp interactions. 7A stands for "Any Halogen".

The I···cg contact is defined as "inter-molecular" and "distance range: 2.5 to 4.5 Å" A total of 23 interactions of this nature have been identified.
